# Optimization of Olive Paste Acidification with Ascorbic and Malic Acids via RSM to Maximize Oil Phenolic and Volatile Composition

**DOI:** 10.3390/foods15122214

**Published:** 2026-06-19

**Authors:** Raul Peralta, Alfonso Manuel Vidal, Francisco Espínola, María Teresa Ocaña, Manuel Moya

**Affiliations:** 1 Department of Chemical, Environmental and Materials Engineering, University of Jaén, Paraje Las Lagunillas, 23071 Jaén, Spain; rpgarcia@ujaen.es (R.P.); fespino@ujaen.es (F.E.); 2 Institute of Biorefineries Research (I3B), University of Jaén, Paraje Las Lagunillas, 23071 Jaén, Spain; amvidal@ujaen.es; 3 Department of Didactics of Sciences, University of Jaén, Paraje Las Lagunillas, 23071 Jaén, Spain; mocana@ujaen.es

**Keywords:** ascorbic acid, malic acid, secoiridoids, antioxidant capacity, photosynthetic pigments, quality parameters

## Abstract

Phenolic compounds, particularly secoiridoids derived from oleuropein and ligstroside, are the main determinants of the antioxidant capacity and health-promoting properties of virgin olive oil, yet their content is strongly affected by processing conditions. This study aimed to enhance phenolic enrichment in Picual olive oil through mild acidification of the paste. Four olive samples were processed under a Central Composite Design varying malaxation time (40–80 min), acid concentration (0.02–0.08 mol/kg paste), and acid type (ascorbic or malic), across two maturity indices (MI) per acid, and evaluated by Response Surface Methodology. Ascorbic acid outperformed malic acid for most of the evaluated responses, with the majority of the monitored parameters exhibiting progressive improvements with increasing acid concentration across the tested range. Extraction efficiency reached 75.8–80.0%, increasing with ripening, malaxation time, and acid dose. Acidification did not affect standard quality parameters but enhanced pigment retention (up to 18.9 mg/kg carotenoids; 28.9 mg/kg chlorophylls) and selectively increased oleuropein- and ligstroside-derived secoiridoids. Antioxidant capacity correlated with phenolic content, reaching 1177.9 µmol Trolox equivalents/kg at high acid concentration and medium–high malaxation times. The optimal acid dose depended on MI, with higher doses favoring riper fruit. Overall, in the Picual cultivar, mild acidification is an effective strategy to enrich the antioxidant fraction of olive oil without compromising its quality.

## 1. Introduction

Olive oil is a staple of diets worldwide, particularly in Mediterranean regions, which account for the major share of global production and consumption. It is widely recognized for its outstanding nutritional quality and health-promoting effects [1]. Its composition is characterized by a high content of unsaturated fatty acids—predominantly oleic acid—alongside a diverse range of bioactive constituents, including phenolic secoiridoids, lignans, tocopherols, and phytosterols. These compounds, together with its distinctive sensory profile, distinguish olive oil as a unique source of dietary fat [2]. The phenolic profile and overall quality of olive oil are shaped by multiple factors, including geographical origin, soil characteristics, climate, cultivation practices, olive variety, fruit maturity, and processing conditions [3,4]. Among the minor constituents, phenolic compounds are particularly significant due to their antioxidant capacity (AC). They play a major role in determining the stability, bitterness, and pungency of virgin olive oils.

Key bioactive molecules such as tyrosol, hydroxytyrosol, and their secoiridoid derivatives have been linked to a wide range of health benefits. These include protective effects against cardiovascular disorders, inflammation, microbial infections, certain types of cancer, and oxidative stress at the cellular level [5,6,7,8,9]. In recognition of these benefits, the European Commission has approved a health claim indicating that “olive oil polyphenols contribute to the protection of blood lipids from oxidative stress,” provided that a minimum daily intake of 20 g of olive oil containing at least 250 mg/kg of polyphenols—especially hydroxytyrosol and its derivatives—is consumed [10].

**Figure 1:**
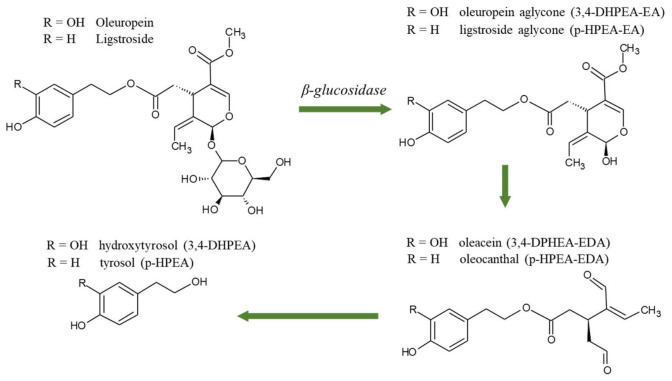
Enzymatic hydrolysis of oleuropein and ligstroside yields secoiridoids, ultimately forming hydroxytyrosol and tyrosol.

Secoiridoids represent the predominant class of phenolic compounds in olive oil, accounting for approximately 80% of the total phenolic content. These molecules are formed during oil extraction: enzymatic release occurs during milling, followed by the hydrolysis of oleuropein and ligstroside during malaxation. The principal secoiridoids are oleacein (3,4-DHPEA-EDA) and oleocanthal (p-HPEA-EDA), accompanied by their respective aglycone precursors, 3,4-DHPEA-EA and p-HPEA-EA [11]. Upon degradation, oleacein and oleocanthal yield hydroxytyrosol and tyrosol, respectively (Figure 1). Among the various antioxidants present in olive oil, hydroxytyrosol (3,4-DHPEA) and its secoiridoid derivatives (3,4-DHPEA-EDA and 3,4-DHPEA-EA) are considered the most effective, exhibiting higher AC than α-tocopherol [12]. Additional phenolic constituents found in virgin olive oil include phenolic acids (e.g., vanillic, ferulic, and p-coumaric acid), lignans (such as pinoresinol), and flavonoids (including luteolin and apigenin) [13,14].

The phenolic and volatile composition of olive oil is primarily influenced by the enzymatic activity inherent to the olive fruit, which is modulated by factors such as geographic origin, ripening stage and water availability. In addition, the technological parameters applied during oil processing have a substantial impact on these chemical profiles [15,16,17,18]. The milling and malaxation steps are particularly critical in determining the final secoiridoid content. Carefully controlled malaxation conditions and lower milling temperatures have been linked to improved preservation of these bioactive phenolics. Optimal processing conditions—such as limited oxygen exposure, slightly acidic pH and malaxation temperatures between 25 and 27 °C—promote the activity of endogenous enzymes, including β-glucosidase and lipoxygenase. This enzymatic activity enhances the formation of both volatile compounds and secoiridoids [19,20]. In contrast, advanced fruit ripening stimulates polyphenol oxidase activity, which contributes to a decline in oleocanthal, oleacein, and their related derivatives.

Both oleacein and oleocanthal exhibit potent antioxidant and anti-inflammatory activities, offering protective effects against hypertension and inflammatory diseases [21,22,23]. Furthermore, they display cardioprotective properties, improve cognitive function in Alzheimer’s disease, and show therapeutic potential against cancer and neurodegenerative disorders [24,25,26,27]. Given this broad spectrum of health-promoting benefits, several studies have focused on developing techniques to isolate and concentrate these secoiridoids from olive oil [28,29,30,31,32]. In this study, to enhance the concentration of these compounds, two acids were evaluated. Ascorbic acid (vitamin C) is a water-soluble organic compound with poor solubility in fats and oils. It is authorized as a food additive under the designation E300 and is widely recognized for its antioxidant properties. Owing to its strong reducing capacity, it is frequently utilized in aqueous systems, oil-in-water emulsions, and oleogels. However, it is not soluble in refined oils or oils devoid of moisture [33,34,35]. On the other hand, malic acid is a naturally occurring dicarboxylic organic acid highly abundant in apples and other fruits, contributing to their characteristic tart flavor. It is highly soluble in water but insoluble in fats and oils. Although its antioxidant activity is lower than that of ascorbic acid, malic acid can enhance cellular metabolism and energy production. It is classified as a food additive under the designation E296 and is widely used in the food industry as an acidulant, flavor enhancer, and pH regulator [36,37].

It is important to emphasize that the addition of organic acids during the extraction process yields a product that deviates from the traditional regulatory definition of “Virgin Olive Oil”. Rather than aiming to obtain a conventional product, the objective of this study is to continue a previous investigation by Peralta et al. [38] in order to obtain the best conditions to develop a functional olive-based lipid matrix or an enriched olive oil in bioactive phenolic compounds. By prioritizing the enhancement of phenolic stability and extraction yield, this approach targets the high-value functional food market, where the incorporation of endogenous organic acids serves as a clean-label technological intervention rather than a traditional milling practice. The research will identify the most effective acid, its optimal concentration and the best malaxation time for improving the studied responses. These key responses are quality parameters, including individual and total secoiridoid content, overall phenolic composition, volatile compound profile, AC and extraction efficiency.

The implementation of central composite designs and Response Surface Methodology (RSM) is a well-established and robust approach for identifying optimal processing conditions in olive oil technology. Recent literature has successfully employed these statistical tools to evaluate innovative extraction techniques, such as high-frequency ultrasound [39,40], and to optimize the use of physical processing aids like CaCO_3_ to improve extraction yields while preserving the oil’s chemical integrity [41]. Moreover, RSM has proven essential in refining malaxation parameters aimed at enhancing the volatile fraction and overall sensory quality [42]. Building upon these established experimental foundations, the present study extends the application of these statistical models to the specific interaction between fruit ripening stages and the addition of selected organic acids. The novelty of this approach lies in the structured modeling framework provided by RSM, which enables not only the estimation of main factor effects but also the identification of significant interactions and non-linear responses that cannot be adequately captured using classical one-factor-at-a-time experimental strategies. Moreover, it reduces the number of experimental runs that are needed. However, RSM relies on the assumption that the fitted models adequately represent the experimental domain, which may limit its predictive power when conditions fall outside the studied range.

While the previous study [38] evaluated ascorbic, citric, and acetic acids at fixed concentrations (1%, 2%, and 4% *w*/*w*) using olives at a single, low maturity index (MI 1.4)—identifying ascorbic acid as the most effective treatment—the present work substantially expands upon those findings. Specifically, malic acid was introduced as a novel candidate for direct comparison with ascorbic acid. Furthermore, the discrete-dose screening strategy was replaced by a RSM approach, enabling the optimization of both acid concentration and malaxation time over a continuous experimental range to capture potential interactions and non-linear effects. Additionally, a second, more advanced maturity index was incorporated to evaluate how fruit ripeness influences the effectiveness of the acidification strategy. The resulting oils, characterized by an enhanced content of bioactive phenolic compounds, offer valuable opportunities for developing functional foods, although they cannot be classified as virgin olive oil under current regulatory frameworks.

Based on these considerations, the present study was designed to test the hypothesis that the mild acidification of olive paste with food-grade organic acids enhances the enzymatic release and subsequent stabilization of secoiridoids and other phenolic compounds, thereby increasing the antioxidant capacity of the resulting olive oil without adversely affecting its standard quality parameters. Furthermore, it was hypothesized that the magnitude of this effect depends on the acid type, its concentration, the malaxation time, and the fruit maturity index. Accordingly, the specific objectives of this study were: (i) to determine which of the two tested acids (ascorbic or malic acid) is more effective in promoting phenolic enrichment; (ii) to optimize acid concentration and malaxation time at different maturity indices using RSM coupled with a central composite design; and (iii) to assess the influence of these factors on extraction efficiency, olive mill wastewater (vegetation water) pH, standard quality parameters, photosynthetic pigments, individual and total phenolic compounds, volatile composition, and the antioxidant capacity of the resulting oils.

## 2. Materials and Methods

### 2.1. Olive Characterization

Olives (*Olea europaea* L., cultivar Picual) were manually collected from rainfed groves located in Jaén, southern Spain, during the 2024–2025 harvesting season. The ripeness of the fruit, expressed as the MI, was evaluated following the protocol established by Vidal et al. [43]. Moisture content was evaluated by drying the ground olive paste at 105 °C for 24 h. Oil content was measured using Soxhlet extraction, following the Commission Regulation [44] with minor methodological modifications. Approximately 10 g of sample were extracted with hexane for 4 h.

### 2.2. Oil Extraction and Water pH

The extraction of olive samples was carried out using a bench-scale Abencor system (MC2 Ingeniería y Sistemas S.L., Sevilla, Spain), in accordance with the protocol detailed by Vidal et al. [43]. Oil extraction was conducted following a Composite Central Design. A constant olive mass of 500 g and a crusher hole diameter of 5.5 mm were used in all assays, and the malaxation temperature was maintained at 25 °C. Detailed information regarding the additional experimental parameters and conditions can be found in Section 2.7.

These parameters were selected based on previous studies that demonstrated their effectiveness in ensuring both industrial feasibility and the preservation of a balanced composition of phenolic and aromatic compounds. The relatively low malaxation temperature was chosen to favor the activity of key endogenous enzymes, particularly lipoxygenase and β-glucosidase [45]. Before malaxation, specific amounts of ascorbic acid (analytical grade, purity 99.0%, Panreac) and malic acid (purity ≥ 99.0%, VWR Chemical) were incorporated in powder form into the olive paste to reach the concentrations shown in Table 1. Both acids are highly water-soluble and practically insoluble in the oil phase. Therefore, once added to the paste—which contains a large aqueous fraction (≈53–62% moisture)—they dissolved in the olive mill wastewater (OMW) and were homogeneously distributed throughout the matrix by the continuous malaxation. The pH was determined in the OMW separated after centrifugation, since a stable and representative reading cannot be obtained directly within the complex paste–oil–water emulsion. Because the added acids equilibrate with this aqueous phase during malaxation, and given that centrifugation is a brief, purely mechanical separation, the OMW pH is representative of the acidic aqueous environment experienced by the paste during processing.

To optimize yield, the malaxed paste was subjected to a two-step centrifugation process, with each cycle lasting 180 s. The collected supernatant was transferred to graduated cylinders. After extraction, the oil underwent 24 h of decantation, followed by filtration through cellulose paper to eliminate residual moisture and organic debris. Finally, the oil was stored at −18 °C under a nitrogen atmosphere until analysis. Extraction efficiency was expressed as the percentage of oil recovered relative to the total oil content in the fruit, as defined by Vidal et al. [43]. The pH of the water samples in the graduated cylinder was measured using a Crison pH meter.

It is pertinent to note that while the Abencor system replicates the fundamental mechanical stages of the olive oil mill (crushing, malaxation, and centrifugation), the yields reported herein represent relative trends rather than absolute industrial values. The use of this standardized laboratory-scale approach was essential to isolate the influence of fruit ripening and organic acid concentration from other industrial confounding variables. Consequently, these results provide a robust scientific foundation and a preliminary optimization screening that necessitates subsequent validation in a commercial olive oil mill setting.

### 2.3. Olive Oil Quality Parameters and Photosynthetic Pigments

The free acidity, peroxide value and extinction coefficients at 232 and 270 nm (K232, K270) were determined in accordance with the procedures outlined by the Commission Regulation [44]. For the necessary measurements of extinction coefficients, an Ultraviolet-Visible (UV–Vis) spectrophotometer (Shimadzu UV-1800, Kyoto, Japan) was used.

The quantification of pigments, specifically chlorophylls and carotenoids, in the oil samples was performed following the method described by Minguez-Mosquera et al. [46]. A 3 g aliquot of oil was dissolved in cyclohexane within a volumetric flask. Measurements were conducted using a UV–Vis spectrophotometer (Shimadzu UV-1800, Kyoto, Japan). Chlorophyll content was determined by absorbance at 670 nm, while carotenoids were quantified at 470 nm. Results were expressed as milligrams of pigment per kilogram of oil.

### 2.4. Phenolic Compounds

Sample preparation was carried out following the protocol established by the International Olive Council (IOC) [47]. The analytical procedure applied for phenolic determination was previously described by Vidal et al. [45].

Quantification of phenolic compounds was conducted using High-Performance Liquid Chromatography (HPLC) (Shimadzu Corp., Kyoto, Japan) equipped with a BDS Hypersil C18 column (Thermo Scientific, Waltham, MA, USA) with dimensions of 25 cm length, 5 μm particle size, and 4.6 mm internal diameter. The mobile phase was composed of water containing 0.2% orthophosphoric acid, methanol, and acetonitrile. Chromatographic conditions included a flow rate of 1 mL/min, an oven temperature of 30 °C, and an injection volume of 20 μL. The UV detector was set to monitor absorbance at 280 nm.

Phenolic compounds were identified by comparing their retention times and spectral data with those of pure standards using an internal standard for improved reliability, and quantification was performed using previously established calibration curves prepared with pure standards. Syringic acid was used as the internal standard for identification and quantification. Calibration curves were prepared for each phenolic compound using concentration ranges adapted to the expected levels of each analyte in the samples. These ranged from lower concentrations below 0.05 ppm for compounds such as vanillin or vanillic acid to upper concentrations exceeding 100 ppm for major secoiridoid derivatives such as oleacein and oleocanthal. In all cases, calibration curves showed excellent linearity, with coefficients of determination (R^2^) higher than 0.997. For ligstroside aglycone and other unidentified phenolics, quantification was performed according to the IOC guidelines. Results were expressed as milligrams of compound per kilogram of oil.

The identification of phenolic compounds was carried out using the following reference standards: vanillic acid, vanillin, luteolin, and p-coumaric acid (Sigma-Aldrich, St. Louis, MO, USA); syringic acid and trans-ferulic acid (Fluka, Milan, Italy); hydroxytyrosol, tyrosol, and apigenin (HPC Standards GmbH, Cunnesdorf, Germany); oleocanthal (Phytolab, Dutendorfer, Germany); and oleacein, oleuropein aglycone, and pinoresinol (TRC, Toronto, ON, Canada).

### 2.5. Antioxidant Capacity

AC was evaluated using the ferric reducing antioxidant power (FRAP) assay, using a Bio-Rad iMark microplate absorbance reader (Bio-Rad Laboratories, Hercules, CA, USA) set to a detection wavelength of 595 nm, following the procedure outlined by Vidal et al. [48]. Calibration was performed using a Trolox standard curve from 0.18 to 1.12 µmol/mL, and results were expressed as Trolox equivalents (TE) per kilogram of oil. Among the available methodologies for assessing antioxidant activity, the FRAP assay was selected due to its high reproducibility and low experimental variability, making it particularly suitable for analyzing the relationship between phenolic antioxidant content and the type and concentration of added acids.

### 2.6. Volatile Compounds

Volatile compounds were analyzed following the protocol described by Vidal et al. [43], which combines headspace solid-phase microextraction (HS-SPME) with gas chromatography coupled to flame ionization detection (GC-FID). Analyses were conducted using a 7890B gas chromatograph (Agilent Technologies, Santa Clara, CA, USA) fitted with a DB-WAXetr capillary column. The SPME fiber, consisting of Carboxen/DVB/polydimethylsiloxane (2 cm length, 50/30 μm film thickness), was supplied by Supelco (Bellefonte, PA, USA). 4-Methyl-2-pentanol was used as the internal standard, and identification of volatile compounds was carried out using 35 external reference standards. Calibration curves were prepared using concentration ranges between 0.096 and 3.2 ppm for most analytes. An exception was trans-2-hexenal, for which the upper calibration limit reached 12 ppm due to its higher expected concentration in the samples. As with phenolics, all calibration curves for volatile compounds exhibited high linearity, with R^2^ values above 0.997 in all cases.

The detected volatiles were classified into three categories: 6-carbon (C6) Lipoxygenase (LOX)-derived compounds, including hexanal, hexanol, (E)-2-hexenal, (E)-2-hexenol, (Z)-3-hexenol, and (Z)-3-hexenyl acetate; 5-carbon (C5) LOX-derived compounds, including 1-penten-3-ol, 1-penten-3-one, and (Z)-2-pentenol; and ethanol. Concentrations were expressed as milligrams of compound per kilogram of olive oil.

### 2.7. Statistical Analysis

To assess the influence of processing parameters on the characteristics of the resulting olive oils, a statistical Design of Experiments coupled with RSM was applied. A rotatable central composite design with five replicates at the central point was employed for each acid, as presented in Table 1. This design facilitates the fitting of second-order polynomial models that include factor interactions. This provides a reliable approximation of the response surface and enables the identification of optimal operating conditions within the experimental range.

The two factors considered were the malaxation time, ranging from 40 to 80 min, and the concentration of the added acid, varying from 0.02 to 0.08 mol of acid/kg of olive paste (mol/kg). These ranges were selected based on preliminary trials and literature criteria to ensure both experimental relevance and practical applicability. In particular, we considered the study by Peralta et al. [38], where ascorbic acid at 1% weight/weight (*w*/*w*) (0.057 mol/kg) proved to be the most effective treatment and where acidified pastes had already been compared with an untreated (acid-free) control. This previously optimal dose was therefore adopted as the reference around which the central level of the present design (0.05 mol/kg) was set. The experimental domain extends to both lower and higher doses, down to a near-zero axial point (0.008 mol/kg) that acts as a minimal-acid baseline. Consequently, a separate untreated control was not repeated here, since the comparison with non-acidified processing was established in [38] and the dose–response relationship captured by the fitted models already accounts for the effect of acid addition across the entire domain.

To ensure a rigorous interpretation of the experimental results, the independent variables were transformed into dimensionless coded levels. This approach allows for a direct comparison of the relative impact of each factor on the response, regardless of their original units. The relationship between the actual values and the coded values is defined as follows:

(1) $$x_{i}=\frac{{2X}_{i}-(a+b)}{b-a}$$

where *x_i_* is the coded factor of the independent variable, *X_i_* is the natural (actual) value of the factor, and *a* and *b* are the minimum and maximum limits, respectively, of the experimental interval. Each factor was assessed at five levels (−α, −1, 0, +1, +α), where level 0 represents the center point of the experimental domain, ±1 the factorial points, and ±α the axial points (Table 1).

The final experimental matrix consisted of 13 randomized runs, including factorial, axial, and central points, as detailed in Table 1. The following response variables were measured: extraction efficiency, pH, quality parameters, photosynthetic pigments, phenolic content, AC, and volatile compounds. The mathematical models were developed using coded factors to ensure that the regression coefficients reflect the relative importance of each independent variable independently of their original units or scales. By transforming the natural variables into a dimensionless range [−1, 1], the direct comparison of the magnitudes of the effects is facilitated, and the numerical stability of the least-squares estimation is enhanced. This approach follows the standard practices for RSM as it allows for a more intuitive interpretation of the synergistic and antagonistic interactions between factors.

A quadratic model was fitted to each response to describe their variation as a function of the two factors, as follows:

(2) *Y* = *β*_0_ + *β*_1_ *A* + *β*_2_ *B* + *β*_12_ *A B* + *β*_11_ *A*^2^ + *β*_22_ *B*^2^ ± *ɛ*

In this equation, *A* and *B* represent the coded factors of malaxation time and acid concentration, respectively. In the model, *Y* represents the response or dependent variable. *β*_0_ is the constant term, representing the value of the response at the center point. *β*_1_ and *β*_2_ are the linear coefficients (first-level terms), while *β*_12_ is the interaction coefficient between factors. The quadratic coefficients are *β*_11_ and *β*_22_, and *ɛ* is the residual standard deviation of the model. The interaction and quadratic terms are considered second-level terms. All models in RSM are hierarchical, meaning that any model containing a second-level term must also include the corresponding first-level term, even if it is not statistically significant (*p*-value > 0.05). Model selection was exclusively based on two criteria: statistical significance (*p*-value < 0.05) and a non-significant lack of fit (*p*-value > 0.05). Consequently, all presented models meet these standards and only include terms that were either statistically significant or required for hierarchical modeling.

When interpreting models based on coded factors, the independent term (*β*_0_) denotes the response value at the center point of all factors, which serves as the reference point for drawing the response surface. The linear coefficients (first-level terms) are the primary determinants of the response surface shape.

These linear effects can be modified by higher-level terms (interaction and quadratic coefficients), with their influence becoming more pronounced as the surface approaches the experimental extremes. Specifically, if the absolute values of the second-level coefficients are equal to or greater than those of the first level, they can significantly alter the surface. Conversely, when second-level coefficients are small, their impact is minimal.

If the linear coefficients are high and the higher-level coefficients are low, the linear terms practically define the response surface. However, when the converse is true, the surface exhibits significant torsion and/or curvature corresponding to the magnitude of the higher-level coefficients.

All statistical analyses were performed using StatGraphics Centurion version 19.1.2 (Statpoint Technologies, Inc., Warrenton, VA, USA). The adequacy of the models was determined using the coefficient of determination (R^2^) and the coefficient of variation (CV), the latter of which is defined as the percentage relative error of the model’s prediction with respect to the mean of the experimental data.

## 3. Results and Discussion

### 3.1. Characterization of Olive Fruit

Olive fruit samples were collected at four distinct ripening stages, corresponding to different MI. The four olive samples were labeled according to the added acid and the order in which they were processed: with ascorbic acid, Asc1 (MI 3.7) and Asc2 (MI 5.2); and with malic acid, Mal1 (MI 4.2) and Mal2 (MI 5.5) (Table 2). For each sample, analyses were performed in quadruplicate to determine moisture content, oil content, and solid matter. The MI values, along with the corresponding means and standard deviations for each parameter, are presented in Table 2.

Table 2 demonstrates that olive moisture content declines with maturation, provided there are no disturbances from external factors (e.g., rainfall). Given that the olives were harvested at an intermediate MI, corresponding to the completion of skin color change, we deduce that lipogenesis is complete. Therefore, the oil content remains stable at approximately 47% on a dry basis across the samples.

### 3.2. Extraction Efficiency

A significant effect of the technological factors on extraction efficiency was evidenced in both the ascorbic acid and malic acid trials (Table 3). The models presented (Table 3) demonstrate that malaxation time is the predominant factor influencing extraction efficiency, exhibiting heightened importance in advanced olive maturation stages. In contrast to time, the influence of the acids is relatively low; nevertheless, a significant positive interaction is observed between the factors for less ripe olives. This positive interaction implies a synergistic effect, where the factors’ influence on the response is amplified by higher values of the companion factor. The quadratic terms do not achieve statistical significance relative to the linear terms but contribute a small convex curvature to the response surfaces.

Based on the models presented in Table 3, the optimal extraction efficiencies for the four samples were determined and are also reported in the same table. The maximum optimal extraction efficiencies ranged between 75% and 80%. In nearly all cases, these maxima were achieved with malaxation times of approximately 80 min and an acid concentration of 0.08 mol/kg. An exception was Sample Asc2, where two negative quadratic terms caused the response surface to curve toward distinct optimum values for each factor.

For Sample Asc2, the quadratic term of factor *A* was approximately half its linear term. Consequently, this curvature—although statistically significant—only marginally affected the factor’s overall influence on the response, defining a maximum around 78 min. In contrast, the quadratic term of factor *B* surpassed its linear term, indicating that the curvature significantly modified the action of factor *B* on the response. For factor *B*, the optimum was determined to be at 0.05 mol/kg.

For both acids, the best extraction performance was achieved at the higher MI. However, the influence of ascorbic acid increased slightly more with increasing MI. Ascorbic acid consistently yielded greater extraction efficiency than malic acid across all trials, strongly suggesting that it represents the superior choice when maximizing yield is the primary objective.

Peralta et al. [38] investigated the effects of adding ascorbic, citric, and acetic acids at 1%, 2%, and 4% (*w*/*w*) to olive pastes, finding that acid addition had a minimal influence on extraction efficiency. Although the highest efficiency was achieved at the lowest acid dose of ascorbic acid (equivalent to 0.057 mol/kg) for an MI of 1.4, that result was not statistically different from that obtained with the 2% dose. These findings are consistent with our previous study, especially considering our optimal concentration of 0.08 mol/kg corresponds to 1.41% (*w*/*w*) for ascorbic acid and 1.07% (*w*/*w*) for malic acid. The overriding conclusion from both investigations is the substantial dominance of malaxation time over acidification in modulating the extraction response.

The pH of the vegetation water was also measured for all trials, following the decantation and oil removal steps. Given the similarity of the statistical models obtained across the four samples for each acid, a single, consolidated model was determined for each acid type. These models clearly reflect the inherent difference in acid strength between the two acids used. As expected, time does not influence pH; only acid concentration does. Specifically, pH of the water ranged from 4.62 to 5.02 for ascorbic acid, but exhibited a lower range of 4.08 to 4.71 for malic acid.

### 3.3. Quality Parameters and Photosynthetic Pigments

Despite not meeting the criteria for classification as virgin olive oil under Regulation (EU) No. 1308/2013 [49], owing to the incorporation of processing aids that chemically lower the pH during extraction, the oils were subjected to analysis of the principal quality parameters commonly used in the sector. The quality parameters showed no statistically significant variations related to either the acid dose or the malaxation time. Consequently, the resulting models for each olive sample were non-significant, and only the mean values of all trials should be considered (Table 4). This consistency, however, contrasts with ripening, which did affect these quality responses to varying degrees.

Free acidity, a key indicator of hydrolytic degradation, remained well below the legal threshold for extra virgin olive oil in all treatments. Neither of the two acids modified the acidity throughout the maturation process. Across the Asc1, Asc2, and Mal2 experimental series, the average acidity was 0.11%. The Mal1 series registered a slightly higher average of 0.12%, a difference that was not statistically significant compared to the other series. This consistency indicates that, under the conditions studied, the presence of acids in the olive pastes and the malaxation time do not alter the final free acidity of the oils.

The peroxide value (PV), which reflects the primary oxidation state of the oil, showed no appreciable change with acid addition. Instead, the PV was primarily affected by olive ripening, though the resulting differences were not statistically significant. Specifically, oils from less mature olives (lower MI) exhibited a PV ranging from 0.25 to 0.29 mEq O_2_/kg, whereas those from more mature olives (greater ripeness) ranged between 0.30 and 0.39 mEq O_2_/kg. This suggests that only advanced olive maturation contributes to a marginally higher peroxide value.

Neither the technological factors nor the agronomic factor of fruit ripening was found to exert a statistically significant influence on K232 (an index of early oxidation) or K270 (an index of advanced oxidation). Across the four olive samples, K232 ranged minimally between 1.20 and 1.28, while K270 showed extreme values between 0.04 and 0.10. If any trend is discernible, both indices showed a slight decline with the progression of olive ripening.

The carotenoid and chlorophyll contents followed the expected downward trend with ripening due to the progressive degradation of these compounds. Notably, oils treated with ascorbic acid appeared to retain higher levels of pigments than those treated with malic acid at similar olive maturation stages.

For ascorbic acid-treated oils, the maximum values were achieved at MI 3.7 (Asc1), reaching 18.89 mg/kg and 28.94 mg/kg for carotenoids and chlorophylls, respectively. These values subsequently decreased to 14.21 mg/kg and 17.41 mg/kg at MI 5.2 (Asc2). In comparison, oils obtained with malic acid exhibited maximum carotenoid and chlorophyll values in the Mal1 series of 15.93 mg/kg and 22.05 mg/kg respectively, which then declined significantly to 10.25 mg/kg and 9.70 mg/kg for the Mal2 series.

Quantitatively, the loss of these pigments with ripening was markedly sharper in the presence of malic acid. Chlorophyll content decreased by 40% in the ascorbic acid-treated oils, compared to a 56% reduction in those treated with malic acid. Similarly, carotenoid content decreased by approximately 25% with ascorbic acid versus 35% with malic acid. This behavior can be attributed to the more pronounced pH decrease induced by malic acid compared with ascorbic acid. Andrés-Bello et al. [50] reported that the most common transformation of chlorophyll is the loss of its central magnesium atom to yield pheophytin, a process wherein the magnesium is replaced within the porphyrin ring by two protons (H^+^)—a reaction favored in an acidic medium. In agreement with this, Acuña et al. [51] observed a greater degradation of chlorophylls (*a* and *b*) and carotenoids when these pigments were maintained in lettuce juice at an acidic pH (4.5) than in a control or a basic medium (pH 7.5), confirming that the conversion of chlorophylls into pheophytins is intensified under acidic conditions.

A study by Vidal et al. [52] on a Picual traditional cultivar with MI fruit values between 1.7 and 1.9, established models with maximum chlorophyll and carotenoid contents of 32.5 and 13.1 mg/kg, respectively. For a considerably lower MI, these values only slightly exceeded the chlorophyll content of our optimum Asc1, whereas carotenoid levels were lower than in all our models except Mal2.

On the other hand, Espínola et al. [53] conducted a study on wild Spanish olive trees known as “acebuche”, using fruits with an MI of 3.5—similar to our case—and obtained models with maximum values of 51.5 mg/kg for chlorophylls and 24.8 mg/kg for carotenoids. These notably high contents were influenced by the cultivar and for a malaxation temperature of 40 °C.

In summary, the addition of acid does not modify the quality parameters of the oils, a finding consistent with Peralta et al. [38]. All measured parameters remained well below established legal limits. The only distinction between the two additives is that ascorbic acid proved superior to malic acid in promoting the retention of pigmentation, showing consistently higher recovery across all trials at similar olive ripeness stages.

### 3.4. Phenolic Content and Antioxidant Capacity

The phenolic fraction of olive oil is predominantly composed of secoiridoid derivatives originating from the hydrolysis of oleuropein and ligstroside (Figure 1). Among these, oleuropein-derived compounds are consistently more abundant than those derived from ligstroside across all oil samples. In particular, oleacein (3,4-DHPEA-EDA) and oleocanthal (p-HPEA-EDA) were identified as the major phenolic constituents, whereas their corresponding aglycones—oleuropein aglycone (3,4-DHPEA-EA) and ligstroside aglycone (p-HPEA-EA)—were present at comparatively lower concentrations.

Given that secoiridoids constitute the main contributors to AC and are associated with well-documented health benefits, this study focused primarily on evaluating the effect of acidification on their formation and preservation. This targeted approach allows for a more mechanistic interpretation of the factors governing phenolic composition, while still considering the overall phenolic content and AC as integrative responses.

The effect of acidification on phenolic composition can be explained by its impact on the enzymatic balance governing both the formation and degradation of secoiridoids in olive paste. The addition of organic acids reduces the pH of the system to moderately acidic values (approximately 3.9–5.2, depending on the acid type, dose, and fruit maturity), thereby altering the activity of key enzymes involved in phenolic metabolism. β-Glucosidase, which catalyzes the hydrolysis of secoiridoid precursors into their bioactive forms, exhibits optimal activity in a slightly acidic pH range, typically around 5.0–5.5 in olive fruit [17,54,55]. Under these conditions, sufficient enzymatic activity is maintained to promote the formation of phenolic compounds.

In contrast, oxidative enzymes such as polyphenol oxidase (PPO) and peroxidase (POD), which are responsible for phenolic degradation, show optimal activity at higher pH values (generally between 6 and 7) and are progressively inhibited as the pH decreases [17,56]. In particular, PPO is highly sensitive to acidic environments, where its catalytic efficiency is significantly reduced due to conformational changes and decreased substrate affinity, potentially leading to partial denaturation at very low pH values (<3) [54]. As a result, acidification acts as a technological strategy that shifts the enzymatic equilibrium toward phenolic formation over degradation. This dual effect—preserving β-glucosidase activity while inhibiting oxidative pathways—favors the accumulation of secoiridoids and related compounds in the oil. In addition, the milder pH decrease induced by ascorbic acid relative to malic acid (olive mill wastewater pH 4.6–5.0 vs. 4.1–4.7) remains sufficient to inhibit oxidative enzymes while keeping the medium closer to the abovementioned optimal pH range for β-glucosidase, which further favors the net accumulation of secoiridoids under ascorbic acid treatment.

In agreement with this mechanistic framework, the phenolic compounds and the AC of the oils were strongly influenced by the factors investigated. The fitted models showed high coefficients for both linear and quadratic terms, indicating a pronounced and non-linear response of these variables. Among the studied factors, acid concentration (B) consistently exerted the greatest influence on the different responses (Table 3 and Table 5), highlighting its central role in modulating the enzymatic balance between phenolic formation and degradation.

Across most of the models, a clear maturity-dependent pattern emerges for the effect of A. In these models, at low MI, A generally appears with a positive coefficient, indicating that longer mixing favors the release or preservation of phenolic compounds. However, as ripening advances, this effect tends to reverse: positive coefficients become weaker (e.g., oleocanthal models from +16.01 in Asc1 to +3.22 in Asc2) or even turn negative as seen in the phenolic content response (e.g., from +6.05 in Mal1 to −6.01 in Mal2). At the same time, negative coefficients become stronger, as noted in the oleacein models.

This behavior can be attributed to the changes that occur in the fruit as ripening advances. As olives reach advanced stages of ripening (beyond the final stages of skin color change), the progressive degradation of cellular structures facilitates the rapid partitioning of phenolic compounds into the aqueous phase, thereby reducing the malaxation time required for their transfer into the oil. Under these conditions, prolonged malaxation does not substantially improve phenolic extraction; rather, it promotes their degradation. This phenomenon is further intensified by the reduced levels of endogenous antioxidants in ripe fruits, which weakens the system’s ability to mitigate oxidative processes. Consequently, extended malaxation enhances the activity of oxidative enzymes and accelerates phenolic oxidation, ultimately resulting in a net decline in phenolic content. In contrast, for olives at intermediate ripening stages (MI 2–4), the opposite behavior is observed. At these stages, cellular structures are still sufficiently intact to limit the immediate release of phenolic compounds, and longer malaxation times enhance their diffusion from the aqueous phase into the oil as indicated by the models in Table 3 and Table 5.

In contrast, the effect of B shows the opposite trend to that detected for A. At lower MI values, factor B (acid concentration) typically exhibits negative coefficients (e.g., in phenolic content models, Table 3) or marginally positive coefficients (e.g., in oleacein models, Table 5). This suggests that, within the tested range, the presence of the acid in the pastes is significant for the response, but its concentration is not the driving factor. However, as maturity progresses, the coefficients tend to increase across all models, indicating that the influence of acid concentration (B) on the response becomes statistically significant. Specifically, positive coefficient values become dramatically more pronounced (e.g., in the AC models, the value shifts from +1.18 in Asc1 to +113.25 in Asc2, Table 5), and even some initial negative effects transition to positive ones.

Beyond the first-level terms, second-level terms that significantly reshape the response surface—such as factor interactions—must be rigorously evaluated in response surface models. In the Asc1 series models for oleuropein derivatives, the positive interaction coefficient significantly surpasses the magnitude of the linear coefficient for Factor B (acid concentration). This interaction renders the influence of B highly significant at extended malaxation times (e.g., +1.83 + 14.55 = +16.38) yet strongly negative at short malaxation times (e.g., +1.83 − 14.55 = −12.72). A corresponding finding is verified in the AC data.

Conversely, for the Mal1 series, the negative interaction coefficient is substantially larger than the linear coefficients of both factors. This relationship implies that the linear influence of one factor is dramatically modulated by the level of the other factor. For example, in the oleuropein model, the influence of B changes from a strong positive at A = −1 (+21.14 + 32.49 = +53.63) to a negative at A = +1 (+21.14 − 32.49 = −11.35). This symmetrical modulation is also evident when examining the limits of B relative to Factor A, and is repeated in the AC response.

Differences in the optimal acid doses across models at different MI can be attributed to the physiological and biochemical changes occurring during fruit maturation. These include a reduction in phenolic precursors, a decline in the activity of key biosynthetic enzymes—such as phenylalanine ammonia-lyase and β-glucosidase—and an increase in oxidative enzyme activity (PPO and POD) [19,57]. Consequently, the phenolic fraction becomes more susceptible to degradation, which in turn enhances the relative effectiveness of acidification treatments in preserving and increasing phenolic content and AC under advanced ripening conditions. In line with this, all measured responses showed a general decrease in their concentration in the oils as maturity increased, regardless of the acid applied.

Regarding total phenolic content, the highest levels (501.43 mg/kg for Asc1 and 440.30 mg/kg for Mal1) were achieved using the least mature olives, coupled with extended malaxation times and low acid concentration. As ripening progresses, a general decline in phenolic compounds is observed in the resulting oils. The optimal maxima for total phenolic content decrease in magnitude (296.40 mg/kg for Asc2 and 217.88 mg/kg for Mal2) and shift toward conditions characterized by high levels of Factor B (acid concentration) and insignificant or low levels of Factor A (malaxation time). The results consistently demonstrate that ascorbic acid leads to higher phenolic content than malic acid, indicating that it is the more effective option for maximizing phenolic levels through olive paste acidification.

For the oleacein response, optimal values were achieved at the highest acid concentration in all models except for Asc1. In the Asc1 series, the quadratic coefficient of Factor B (acid concentration) exceeds the linear coefficient, making the curvature of the response surface dominant over its linearity. Since the quadratic term is negative, this results in a convex surface with an optimum located within the experimental range (0.06 mol/kg). Regarding malaxation times, the models show variable optimal times, indicating the low influence of this factor on the response. This low influence is further verified by comparing the small coefficients of Factor A to the independent term.

**Figure 2:**
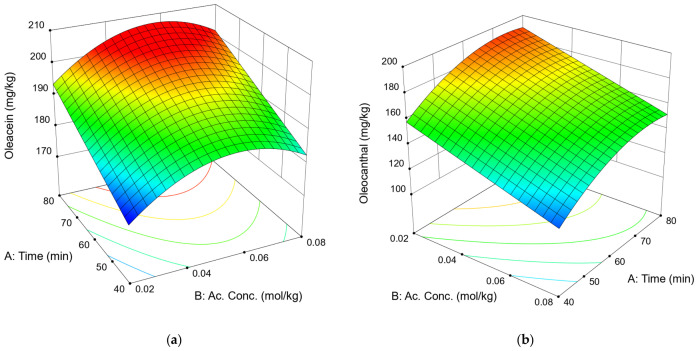
Estimated response surfaces for the content of oleacein (**a**) and oleocanthal (**b**) as a function of malaxation time (A) and acid concentration (B) in sample Asc1 (ascorbic acid, MI 3.7). In each panel, the axes were arranged to allow an adequate visualization of the corresponding surface and therefore differ between panels (**a**,**b**).

In contrast, oleocanthal exhibited an entirely different optimum: the lowest acid concentration combined with high malaxation times. This behavior suggests a formation pathway more dependent on malaxation time and a lower susceptibility to oxidative and degradative processes compared to other secoiridoids. These contrasting behaviors are illustrated by the response surfaces in Figure 2: oleacein content reaches a maximum at an intermediate acid concentration (≈0.06 mol/kg), whereas oleocanthal exhibits a steady increase toward the lowest acid concentration. These findings are consistent with previous studies reporting increases in oleocanthal concentration with longer malaxation times [45,50].

The concentration of oleacein and oleocanthal in the oil depends on the balance between their β-glucosidase-mediated synthesis and their oxidative degradation [17,58,59]. The experimental results showed that oleacein, likely due to its catechol structure (i.e., two hydroxyl groups attached to the benzene ring) [60] and its high antioxidant potential, exhibits greater susceptibility to degradation during malaxation than oleocanthal. These findings are consistent with observations reported by authors such as Olmo-Cunillera et al. [61] and Gómez-Rico et al. [20], who described similar kinetic behaviors for specific secoiridoids, highlighting that oxidative degradation acts more selectively on hydroxytyrosol derivatives. This phenomenon explains why oils subjected to prolonged malaxation retain their pungency intensity while exhibiting a marked reduction in antioxidant capacity.

For oleuropein derivatives and AC (Table 5), the optimum values consistently occurred at the highest acid concentration used (0.08 mol/kg), paired with malaxation times that were either variable or negligible within the experimental range. As fruit maturity increased, both AC and the concentration of oleuropein-derived compounds declined; however, this reduction was markedly less pronounced in oils treated with ascorbic acid compared to those treated with malic acid. Specifically, decreases of approximately 22% were noted under ascorbic acid treatments, whereas reductions exceeded 50% with malic acid, highlighting the greater protective effect of the former. Therefore, to maximize the content of these bioactive compounds, the preferred strategy is to use low-ripeness olives with an ascorbic acid content of 1.4% (*w*/*w*).

The optimal values for specific phenolic compounds are presented in Supplementary Table S1. Secoiridoids mirrored the overall trend of total phenols, with the Asc1 model yielding the highest optimum. However, a divergence was noted at high MI, where these models became independent of malaxation time. The aggregate content of phenolic alcohols decreased with advancing MI, stabilizing near 7–8 mg/kg for both acid treatments. Despite this overall trend, their individual responses were distinct: optimal hydroxytyrosol yields required the highest acid concentrations, while tyrosol optima were found at the lowest concentrations. This observation highlights that increasing acid concentration is advantageous for hydroxytyrosol recovery from high MI olives but unfavorable for tyrosol. The optimal conditions for phenolic acids and derivatives, and lignans were defined by the lowest MI within the experimental range, combined with a high acid concentration and, typically, minimal malaxation times. Conversely, maximum flavonoid levels were achieved under conditions of low acid concentration and short malaxation times.

Peralta et al. [38] reported a positive, group-specific influence of acidification on the phenolic content and AC of oils with optimal results achieved at the lowest ascorbic acid concentration tested (1% *w*/*w*, 0.057 mol/kg equivalent). To further characterize this optimum, the experimental design for the current study was centered at 0.05 mol/kg, establishing a range of acid concentration from 0.02 to 0.08 mol/kg. Both studies align in concluding that the highest total phenolic content is achieved under conditions of low acid concentration (the minimum concentration investigated in both cases) when processing green olives or olives at intermediate ripening stages (MI 2–4).

In the model results, ascorbic acid has proven to be superior to malic acid in increasing the content of phenolic compounds in the oil. This superior behavior can be attributed to the mechanism described by Zhou et al. [56], whereby, in addition to lowering the pH of the medium and partially inhibiting the activity of oxidative enzymes, ascorbic acid acts both as an antioxidant and as a reducing agent capable of regenerating phenolic compounds from the quinones formed during oxidation processes, thereby delaying their irreversible degradation. This additional mode of action of ascorbic acid may explain the results obtained in Peralta et al. [38] and in the present study, in which this acid exhibits greater effectiveness than the other acids evaluated. In addition, the milder pH decrease induced by ascorbic acid relative to malic acid (olive mill wastewater pH 4.6–5.0 vs. 4.1–4.7) remains sufficient to inhibit oxidative enzymes while keeping the medium closer to the optimal pH range for β-glucosidase (≈5.0–5.5), which further favors the net accumulation of secoiridoids under ascorbic acid treatment.

Migliorini et al. [62] observed that the addition of antioxidants such as ascorbic acid or citric acid during processing can limit the oxidative degradation of phenolic compounds when olives exhibit mechanical damage or tissue deterioration, with ascorbic acid showing superior performance, in agreement with the results of both the present and previous studies. However, in trials conducted with healthy olives, no significant differences were reported between oils produced with added antioxidants and those without additive incorporation.

Focusing exclusively on the use of ascorbic acid, a clear trend emerges regarding optimal dosage. For olives with an MI of 3.7, the optimum oleacein content was achieved with an acid dose of 0.06 mol/kg. When the MI increased to 5.2, the required optimal dose rose to 0.08 mol/kg. This finding aligns with previous work, where the optimum for olives at an MI of 1.4 was determined to be 1% (*w*/*w*) (a value similar to that found for the intermediate ripening stage in the present study). The behavior evidenced for oleocanthal is the inverse of that for oleacein: the maximum oleocanthal content is only achieved when the minimum acid concentration is applied. This finding is consistent with the results reported in the previous article.

Within the secoiridoid group, oleuropein derivatives exhibit higher reactivity and greater susceptibility to oxidation due to their catechol-type structure, characterized by the presence of two –OH groups attached to the aromatic ring [58], as previously noted. In contrast, ligstroside derivatives contain only a single phenolic hydroxyl group, which reduces their susceptibility to oxidation. PPO catalyzes the oxidation of these phenolic groups, leading to the formation of highly reactive quinones, thereby decreasing the concentration of these compounds in the oil. In this context, inhibition of PPO activity more effectively promotes the preservation of oleuropein derivatives compared to ligstroside derivatives.

A previous study of Peralta et al. [63] employed RSM using talc and determined a maximum phenolic content of 437.62 mg/kg and a maximum AC of 1333.13 µmol TE/kg for olives with an MI of 3.9. This phenolic content figure is lower than the maxima achieved in the current Asc1 and Mal1 experimental series (501.43 and 440.30 mg/kg, respectively) at comparable MI. In contrast, the AC was higher than the values predicted in our current models, likely reflecting the influence of agronomic factors on final oil composition. Conversely, the maximum phenolic content and AC reported in Peralta et al. [38] was 540 mg/kg and 1712.53 µmol TE/kg for oils obtained with 1% (*w*/*w*) acid but at a significantly lower MI of 1.4. This disparity is primarily explained by the different agronomic ripening stages of the olives and by the influence of various agronomic factors that vary in their impact from year to year.

The optimal yields achieved in the current study for oleacein (207.25 mg/kg) and oleocanthal (191.43 mg/kg) using both acids at a lower MI are significantly higher than the corresponding optima reported by Peralta et al. [63] using talc (180.85 mg/kg for oleacein at MI 2.8 and 124.81 mg/kg for oleocanthal at MI 1.8). In contrast, Peralta et al. [38], who employed a 0.057 mol/kg (eq 1% *w*/*w*) ascorbic acid concentration, reported a higher mean optimal value for oleacein (237.58 mg/kg). However, their maximum oleocanthal value (91.39 mg/kg) was substantially lower than the maximum value achieved in the present investigation.

In olive oil production research, the incorporation of Arbequina leaves during milling was determined to cause a statistically significant elevation in the oil’s phenolic content [64]. Conversely, a subsequent study involving the addition of fresh Arbequina and Santulhana leaves to the mill to increase phenolic content reported a reduction instead. Marx et al. [65] postulated that this decrease resulted from the partial inhibition of β-glucosidase activity.

### 3.5. Oleuropein Derivatives and Antioxidant Capacity

**Figure 3:**
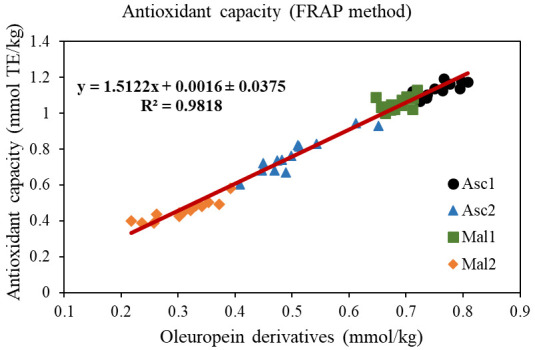
Antioxidant capacity of oleuropein derivatives (hydroxytyrosol, oleacein, and oleuropein aglycone).

To establish the AC of the main oil phenolic compounds (oleuropein derivatives), Figure 3 presents the results from all experimental series, plotting AC against the total moles of hydroxytyrosol, oleacein, and oleuropein aglycone. This molar summation is utilized because the antioxidant effect is dependent on the number of active molecules, regardless of their individual molecular mass.

Although minor constituents such as tocopherols also contribute to the AC of olive oil, their relatively low concentrations indicate that the observed variations are primarily driven by changes in oleuropein-derived secoiridoids and phenolic alcohols, as previously reported by Servili et al. [12]. The experimental data displayed in Figure 3 demonstrate an excellent fit to a straight line, confirming that the relationship holds regardless of variations in malaxation time, acid type, or concentration. From this linear model, a residual antioxidant activity of 0.0016 mmol TE/kg is deduced, representing the baseline antioxidant contribution of other molecules in the oil. Furthermore, the slope reveals a specific AC of 1.5122 mmol TE/mmol for the total oleuropein derivatives. The narrow standard deviation associated with this estimate (shown in the regression equation in Figure 3), together with the low coefficient of variation in the model (CV = 4.40%), confirms the high precision and robustness of the fit. This indicates that the specific antioxidant capacity of the oleuropein derivatives remains essentially constant across the entire range of malaxation times, acid types, and concentrations evaluated.

Based on the premise that the three oleuropein derivatives (shown in Figure 1) possess equal and constant molar AC, the mass-based capacity for each compound was calculated using its molecular mass. These calculated values are: hydroxytyrosol (9.81 μmol TE/mg), oleacein (4.72 μmol TE/mg), and oleuropein aglycone (4.00 μmol TE/mg). These results are slightly lower than those determined by [38], (averaging 4.63 μmol TE/mg for the three molecules) but are higher than the value reported by Vidal et al. [45] (3.87 μmol TE/mg for total phenols).

### 3.6. Volatile Compounds

For volatile compounds, including LOX-derived products and ethanol, the RSM models did not reach statistical significance. For this reason, the corresponding results are presented as trends rather than definitive predictive relationships. These limitations likely reflect the small sample size inherent to RSM designs and the naturally high variability of volatile compounds, which are strongly affected by subtle changes in fruit physiology and processing conditions.

Despite the lack of statistical significance, the mean values provide a useful perspective on the general behavior of LOX-derived volatiles under the different experimental conditions. For both acids, a decreasing trend was observed between the first and second MI studied, suggesting a dependence on both the acid type and fruit ripening stage. For ascorbic acid treatments, the average volatile content decreased from 16.51 mg/kg (Asc1) to 12.88 mg/kg (Asc2), while malic acid treatments showed a reduction from 14.04 mg/kg (Mal1) to 11.79 mg/kg (Mal2). This reduction aligns with the expected loss of enzyme activity and precursor availability during ripening, which limits the formation of the C6 aldehydes and alcohols responsible for the fresh, green aroma of virgin olive oil. In the study conducted by Peralta et al. [63], the optimum value for total LOX in the models of olives with an MI of 3.9—similar in maturity to those in the present study—was 15.87 mg/kg, which is lower than that obtained for Asc1 and higher than that obtained for Mal1.

Although no statistically significant influence of malaxation time or acid dose was established, the recorded decrease across ripening stages is consistent with two trends: early harvest conditions are more favorable for volatile compound preservation, and ascorbic acid appears more effective than malic acid in promoting LOX enzyme activity. This can be explained by the pH dependence of the pathway. LOX displays its highest activity at a slightly acidic pH—with optima reported in the range of 4.0–5.5 [66,67,68]—meaning that mild acidification keeps the medium within or close to this optimal range. Concurrently, the antioxidant action of ascorbic acid further limits the oxidative degradation of the newly formed C6 aldehydes. The highest concentration of volatile compounds in all experimental series was attributed to trans-2-hexenal. The measured concentrations (mg/kg) were 9.94 for Asc1, 8.27 for Asc2, 6.34 for Mal1, and 5.93 for Mal2, highlighting its dominance in the LOX pathway profile.

In the study by Peralta et al. [38], fruits harvested at an MI of 1.4 produced mean Total LOX values of 15.05 mg/kg when the oils were acidified with 1% *w*/*w* ascorbic acid (0.056 mol/kg). The highest values were obtained using 1% citric acid and 4% *w*/*w* ascorbic acid, which yielded 20.02 mg/kg and 17.34 mg/kg, respectively. Similarly, trans-2-hexenal was the most abundant volatile compound, reaching values close to 9 mg/kg under the same conditions that maximized Total LOX in the previous study. In contrast, oils acidified with 1% ascorbic acid showed a mean value of 7.88 mg/kg.

The ethanol content of the oils was analyzed, confirming that malaxation time favors the fermentation of organic matter, leading to greater ethanol accumulation in ripe olives. The statistical influence of time, however, was only noted in the ascorbic acid treatments. The acids exhibited contrasting effects: ascorbic acid has a negative influence, suggesting it delays fermentation, especially at higher doses. In the Asc1 series, ethanol was only detected after 75 min of malaxation at the 0.08 mol/kg dose, but was present at the 40 min mark for doses below 0.03 mol/kg. This sensitivity diminishes in the riper Asc2 series, where minimum ethanol was present immediately at any dose. The maximum and minimum ethanol values (mg/kg) recorded were: 0.87 and 0.00 for Asc1, and 1.87 and 0.59 for Asc2.

In contrast, malic acid showed no significant influence from time or acid concentration, yet these oils yielded the highest ethanol contents (averages: 1.34 mg/kg for Mal1 and 3.36 mg/kg for Mal2). This suggests that malic acid, unlike ascorbic acid, does not appear to limit fermentation. In the previous study by Peralta et al. [38], a decrease in ethanol content was also observed under most acidification conditions, particularly when acidification was carried out with acetic acid and at low doses of ascorbic acid. These results support the hypothesis that certain acids may limit ethanol fermentation.

From a chemical engineering perspective, the inclusion of ascorbic acid was evaluated as a process intensification factor. Although ascorbic acid possesses well-known O_2_ scavenging properties, its role in this study was analyzed as part of a holistic optimization of the extraction process. The significant increase in phenolic and volatile compounds suggests a favorable shift in the extraction equilibrium and enzymatic activity (such as PPO and POD inhibition), likely driven by the synergistic effect of pH modification and antioxidant protection. Future mechanistic studies may be required to decouple these individual pathways; however, for the purpose of functional oil production, the overall enhancement of the lipid matrix remains the primary technological outcome.

### 3.7. Correlation Analysis Among the Response Variables

**Figure 4:**
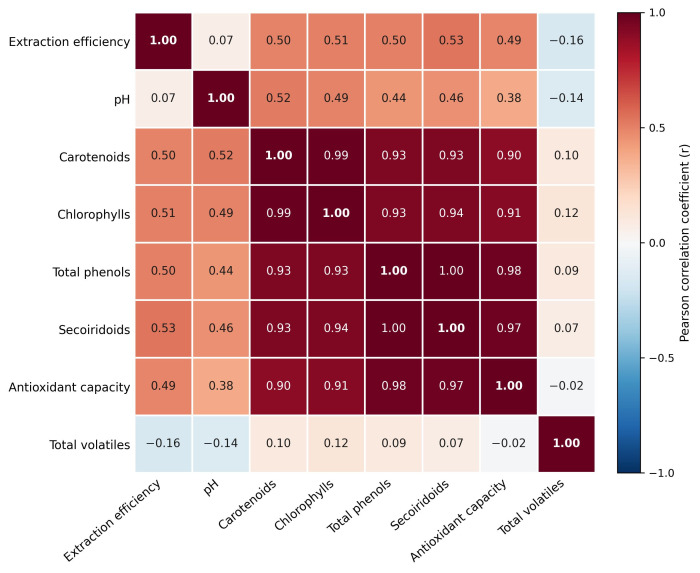
Pearson correlation heatmap among the main response variables across all experimental runs (n = 52; n = 51 for pairs involving carotenoids, chlorophylls or total volatiles, owing to one missing observation in sample Asc2). The color and the value in each cell denote the Pearson correlation coefficient (r), ranging from −1 (blue) to +1 (red).

To unify the interpretation of the dataset, a Pearson correlation analysis was carried out among the main response variables across all experimental runs (Figure 4), revealing a clearly structured pattern. As expected, total phenolic content and secoiridoid content were almost perfectly correlated (r = 1.00), consistent with secoiridoids constituting the predominant phenolic class in these oils. Accordingly, both parameters were strongly associated with antioxidant capacity (r = 0.98 and r = 0.97, respectively), confirming that the secoiridoid fraction is the main determinant of the antioxidant behavior and reinforcing the linear relationship shown in Figure 3. The two photosynthetic pigments were, in turn, almost collinear (carotenoids vs. chlorophylls, r = 0.99), as both are co-extracted and follow parallel trends during fruit ripening.

A less self-evident feature was the strong positive covariation between the pigment fraction and the phenolic–antioxidant block (r = 0.90–0.94). This parallel behavior principally reflects a shared dependence on fruit maturity, since both pigments and phenolics decline as ripening advances. Moreover, the positive association of olive mill wastewater (OMW) pH with the pigment fraction (r ≈ 0.50) is mechanistically consistent with the pH-dependent degradation of pigments discussed in Section 3.3. A lower pH promotes the conversion of chlorophylls into pheophytins and the degradation of carotenoids; thus, the milder acidification induced by ascorbic acid—and the higher residual pH it leaves—favors pigment retention. Extraction efficiency showed moderate positive correlations with pigments, phenols, and antioxidant capacity (r = 0.49–0.53), suggesting that the conditions favoring oil release also promote the co-extraction of these minor constituents. In contrast, the total volatile content was essentially independent of the phenolic and antioxidant fraction (|r| ≤ 0.16), indicating that the lipoxygenase pathway responsible for volatile formation is largely decoupled from the secoiridoid enrichment promoted by acidification.

Finally, it should be noted that this matrix pools the runs of the four sample sets (two acids × two MI); consequently, part of the observed covariation reflects systematic differences between sample groups rather than the response within each individual experimental design. The coefficients should therefore be interpreted as a global, integrative descriptor of the entire dataset.

## 4. Conclusions

This study demonstrates that, for the Picual cultivar, controlled acidification of olive paste, optimized through RSM, is an effective strategy to enhance the phenolic profile and antioxidant capacity of olive oils without compromising conventional quality parameters. Within the experimental domain, malaxation time was the dominant factor for extraction efficiency, whereas acid concentration played a more decisive role in modulating phenolic composition and antioxidant capacity.

Fruit maturity emerged as the primary determinant of oil composition. Within the maturity range studied (MI 3.7–5.5), total phenolics, secoiridoids, pigments, and volatile compounds consistently decreased with advancing ripening. However, acidification—particularly with ascorbic acid—partially mitigated this decline. The optimal technological strategy was maturity-dependent: for lower MI, moderate-to-long malaxation combined with low acid doses maximized phenolic recovery, whereas at higher MI, increased acid concentration and shorter malaxation times were required to preserve bioactive compounds.

Among the acids tested, ascorbic acid consistently outperformed malic acid in promoting extraction efficiency, phenolic enrichment, antioxidant capacity, pigment retention, and preservation of LOX-derived volatiles. These findings indicate that acid type critically influences both enzymatic pathways and the resulting oil composition.

From an applied perspective, harvesting olives before or near the completion of skin color change and applying moderate malaxation (60–80 min) with ascorbic acid at the upper range of the studied concentrations (≈1.5% *w*/*w*) provides the most favorable balance between extraction yield and enrichment in health-related phenolics. Although the resulting product cannot be classified as virgin olive oil under current regulations, this technological approach offers a viable pathway for developing olive-based lipid matrices with enhanced functional properties, supporting innovation in the functional food sector.

Future research should validate these findings at pilot and industrial scales to confirm their practical applicability. It should be noted that the optimization models developed here are valid strictly within the investigated maturity range (MI 3.7–5.5); their extrapolation to other maturity stages was not evaluated. Expanding the study to additional cultivars and broader maturity ranges would help determine the robustness of the proposed optimization strategy. Additionally, complementary, mechanism-based antioxidant assays such as ORAC could be incorporated to characterize the antioxidant potential through a distinct chemical mechanism from the FRAP assay used here. Further mechanistic investigations are also warranted to better disentangle the individual contributions of pH modulation, ascorbic acid O_2_ scavenging properties, enzymatic regulation, and antioxidant protection in the observed enhancement of phenolic and volatile profiles. Such studies would strengthen the scientific basis for the controlled production of phenolic-enriched functional olive oils. In addition, since the phenolic fraction—particularly the secoiridoids responsible for the bitterness and pungency of the oil—is closely linked to sensory perception, future work should incorporate a formal sensory evaluation of the acidified products to establish how the enhanced phenolic content translates into their organoleptic profile.

## Figures and Tables

**Table 1:** Central composite design in terms of coded factor and actual factor.

Run	Time		Concentration	
	Coded Factor A	Actual Factor t (min)	Coded Factor B	Actual Factor c (mol/kg)
1	0	60	+α	0.092
2	0	60	0	0.05
3	−1	40	1	0.08
4	1	80	−1	0.02
5	0	60	0	0.05
6	−1	40	−1	0.02
7	0	60	−α	0.008
8	0	60	0	0.05
9	−α	32	0	0.05
10	1	80	1	0.08
11	0	60	0	0.05
12	0	60	0	0.05
13	+α	88	0	0.05

Level 0 represents the center point of the experimental domain, ±1 the factorial points, and ±α the axial points (α = 1.414).

**Table 2:** Characterization of raw olive samples.

Acid	Label	Maturity Index	Water, %	Oil, %	Solids, %
Ascorbic acid	Asc1	3.7	62.2 ± 0.6 ^a^	17.6 ± 0.2 ^a^	20.2 ± 0.6 ^a^
	Asc2	5.2	53.5 ± 0.6 ^b^	22.5 ± 0.4 ^d^	24.0 ± 0.5 ^b^
Malic acid	Mal1	4.2	61.6 ± 0.7 ^a^	18.4 ± 0.3 ^b^	19.9 ± 0.5 ^a^
	Mal2	5.5	53.5 ± 0.4 ^b^	21.6 ± 0.4 ^c^	24.9 ± 0.7 ^c^
Fisher-LSD			0.9	0.5	0.9

Mean and standard deviation of four replicates. For each column, superscript letters indicate statistical groupings based on Fisher’s least significant difference (LSD) test. Different letters denote statistically significant differences, while identical letters indicate no significant difference at the 95% confidence level.

**Table 3:** Models, in coded factors, statistical parameters, response optimum, and real factor in the optimum.

Response	Label	Model *	R^2^	CV (%)	Optimum	Time (min)	Acid Concentration (mol/kg)
Extraction efficiency (%)	Asc1	72.41 + 3.54A + 1.67B + 3.97AB − 3.13A^2^ ± 0.78	0.990	1.1	78.46	80.0	0.08
	Asc2	74.83 + 11.31A − 1.15B − 6.33A^2^ − 3.51B^2^ ± 1.14	0.996	1.7	79.98	77.9	0.05
	Mal1	72.37 + 4.47A − 2.28B + 3.23AB − 2.69A^2^ ± 1.15	0.953	1.6	75.10	80.0	0.08
	Mal2	64.67 + 8.07A + 3.93B − 0.9AB ± 0.51	0.997	0.8	75.76	80.0	0.08
Total LOX (mg/kg)	Asc1	16.51 ± 0.43	-	2.6	16.51	-	-
	Asc2	12.88 ± 0.33	-	2.6	12.88	-	-
	Mal1	14.04 ± 0.68	-	4.8	14.04	-	-
	Mal2	11.79 ± 0.55	-	4.6	11.79	-	-
Phenolic content (mg/kg)	Asc1	465.39 + 20.57A − 15.47B ± 14.42	0.737	3.1	501.43	80.0	0.02
	Asc2	288.43 + 7.96B ± 6.85	0.425	2.4	296.40	-	0.08
	Mal1	406.11 + 6.05A − 16.25B + 11.89A^2^ ± 7.42	0.893	1.8	440.30	80.0	0.02
	Mal2	200.56 − 6.01A + 11.32B ± 4.1	0.906	2.0	217.88	40.0	0.08

* According to the coefficients of the model (Equation (2)), only significant values are included (*p*-value < 0.05). A is the coded factor of malaxation time (min). B is the coded factor of the acid concentration in the mixture (mol/kg). R^2^ is the coefficient of determination. The model error is the standard deviation. CV is the coefficient of variation.

**Table 4:** Quality Parameters and Photosynthetic Pigments.

Response	Label	Mean	CV
Acidity (%)	Asc1	0.111 ± 0.008	7.65
	Asc2	0.114 ± 0.007	6.16
	Mal1	0.124 ± 0.013	10.37
	Mal2	0.107 ± 0.005	4.26
Peroxide Index (mEq O_2_/kg)	Asc1	0.293 ± 0.015	5.19
	Asc2	0.394 ± 0.030	8.01
	Mal1	0.251 ± 0.011	4.31
	Mal2	0.298 ± 0.085	28.40
K232	Asc1	1.28 ± 0.01	1.12
	Asc2	1.21 ± 0.03	2.88
	Mal1	1.23 ± 0.01	1.19
	Mal2	1.20 ± 0.03	2.78
K270	Asc1	0.104 ± 0.004	3.96
	Asc2	0.074 ± 0.006	8.61
	Mal1	0.087 ± 0.004	4.87
	Mal2	0.040 ± 0.004	10.22
Carotenoids (mg/kg)	Asc1	18.89 ± 1.18	6.24
	Asc2	14.21 ± 0.56	3.97
	Mal1	15.93 ± 0.66	4.16
	Mal2	10.25 ± 0.82	8.05
Chlorophylls (mg/kg)	Asc1	28.94 ± 2.95	10.20
	Asc2	17.41 ± 0.81	4.66
	Mal1	22.05 ± 1.30	5.90
	Mal2	9.70 ± 1.60	16.52

Data are expressed as mean ± standard deviation; CV, coefficient of variation (%), calculated as (standard deviation/mean) × 100.

**Table 5:** Models, in coded factors, statistical parameters, response optimum, and real factor in the optimum.

Response	Label	Model *	R^2^	CV (%)	Optimum	Time (min)	Acid Concentration (mol/kg)
Oleacein (mg/kg)	Asc1	197.28 + 9.53A + 4.08B − 9.28B^2^ ± 3.25	0.930	1.7	207.25	80.0	0.06
	Asc2	117.25 − 4.58A + 24.65B ± 3.3	0.974	2.7	146.48	40.0	0.08
	Mal1	165.58 − 1.85A + 5.42B − 7.98AB + 5.01A^2^ ± 2.04	0.969	1.2	185.83	40.0	0.08
	Mal2	78.29 − 5.13A + 17.91B + 2.7AB − 7.83A^2^ − 2.68B^2^ ± 0.95	0.998	1.3	93.70	56.9	0.08
Oleocanthal (mg/kg)	Asc1	167.07 + 16.01A − 19.02B − 11.99A^2^ ± 6.32	0.948	3.9	191.43	73.3	0.02
	Asc2	88.64 + 3.22A − 5.23B ± 2.98	0.858	3.3	97.09	80.0	0.02
	Mal1	132.23 + 9.89A − 18.89B + 3.89A^2^ ± 2.75	0.988	2.0	164.90	80.0	0.02
	Mal2	61.49 − 3.55B ± 3.05	0.527	4.9	65.03	-	0.02
Hydroxytyrosol + Oleacein + Oleuropein aglycone (µmol TE/kg)	Asc1	756.15 + 18.2A + 1.83B + 14.55AB ± 12.46	0.849	1.7	790.72	80.0	0.08
	Asc2	483.63 − 20.25A + 77.18B + 31.28B^2^ ± 10.1	0.988	2.0	612.33	40.0	0.08
	Mal1	700.54 − 6.96A + 24.14B − 32.49AB ± 10.92	0.857	1.6	764.13	40.0	0.08
	Mal2	312.95 + 53.02B ± 10.68	0.947	3.3	365.00	-	0.08
Antioxidant capacity (µmol TE/kg)	Asc1	1119.23 + 26.14A + 1.18B + 31.35AB ± 17.77	0.809	1.6	1177.90	80.0	0.08
	Asc2	760.2 − 53.27A + 113.25B ± 20.88	0.975	2.7	926.72	40.0	0.08
	Mal1	1041.31 − 22.59A + 17.37B − 36.43AB ± 24.45	0.692	2.3	1117.71	40.0	0.08
	Mal2	463.03 + 37.26B ± 15.63	0.800	3.4	500.29	-	0.08

* According to coefficients of model (Equation (2)), only significant values are included (*p*-value < 0.05). A is the coded factor of malaxation time (min). B is coded factor of the acid concentration in the mixture (mol/kg). R^2^ is the coefficient of determination. The model error is the standard deviation. CV is the coefficient of variation.

## Data Availability

The data presented in this study are available on request from the corresponding author due to privacy.

## Supplementary Materials

The following supporting information can be downloaded at: https://www.mdpi.com/article/10.3390/foods15122214/s1, Table S1: Response optimum and real factors in the optimum for specific phenolic compounds and phenolic groups.

## Abbreviations

The following abbreviations are used in this manuscript:

3,4-DHPEA-EDA	Oleacein
*p*-HPEA-EDA	Oleocanthal
AC	Antioxidant capacity
C5	5-Carbon compounds
C6	6-Carbon compounds
CV	Coefficient of variation
DoE	Design of Experiments
FID	Flame ionization detector
FRAP	Ferric-reducing antioxidant power
GC	Gas chromatography
HPLC	High-Performance Liquid Chromatography
HS	Headspace
IOC	International Olive Council
K_232_	Extinction coefficient at 232 nm
K_270_	Extinction coefficient at 270 nm
LOX	Lipoxygenase
M	Molar
MI	Maturity index
OMW	Olive mill wastewater
POD	Peroxidase
PPO	Polyphenol oxidase
R^2^	Coefficient of determination
RSM	Response Surface Methodology
SPME	Solid-Phase Microextraction
TE	Trolox equivalents
UV-Vis	Ultraviolet–Visible
*w*/*w*	weight/weight
