# Supplementary material for: Optimization of Olive Paste Acidification with Ascorbic and Malic Acids via RSM to Maximize Oil Phenolic and Volatile Composition
Source: Foods. 2026 Jun 19;15(12):2214. doi: 10.3390/foods15122214 (PMC13297711; doi:10.3390/foods15122214)
Supplement: Supplementary file 1 [file foods-15-02214-s001.zip › foods-4361468-supplementary.pdf]

Supplementary Table S1. Response optimum and real factors in the optimum for specific phenolic compounds and phenolic groups

Response	Label	Optimum	Time (min)	Acid Concentration (M)
Hydroxytyrosol (mg/kg)	Asc1	4.58	40.0	0.05
	Asc2	4.61	68.8	0.08
	Mal1	4.29	80.0	0.08
	Mal2	4.77	40.0	0.08
Tyrosol (mg/kg)	Asc1	3.62	80.0	0.02
	Asc2	4.01	80.0	0.02
	Mal1	3.95	40.0	0.02
	Mal2	4.43	40.0	0.02
3.4-DHPEA-EA (mg/kg)	Asc1	53.63	75.5	0.03
	Asc2	43.36	40.0	0.08
	Mal1	51.44	80.0	0.03
	Mal2	24.31	62.2	0.08
p-HPEA-EA (mg/kg)	Asc1	8.12	80.0	0.08
	Asc2	7.99	40.0	0.08
	Mal1	8.41	80.0	0.02
	Mal2	5.20	40.0	0.08
Alcohols (mg/kg)	Asc1	7.73	80.0	-
	Asc2	7.43	-	0.08
	Mal1	7.19	-	-
	Mal2	6.94	-	-
Acids and derivates (mg/kg)	Asc1	2.86	-	0.08
	Asc2	1.90	-	-
	Mal1	2.75	80.0	0.08
	Mal2	2.08	40.0	0.08
Flavonoids (mg/kg)	Asc1	7.88	40.0	0.02
	Asc2	6.64	-	-
	Mal1	7.57	-	0.02
	Mal2	5.02	40.0	0.02
Lignans (mg/kg)	Asc1	22.25	40.0	-
	Asc2	18.91	40.0	0.08
	Mal1	25.15	40.0	0.08
	Mal2	20.66	40.0	-
Secoiridoids (mg/kg)	Asc1	444.22	80.0	0.02
	Asc2	264.84	-	0.08
	Mal1	388.97	80.0	0.02
	Mal2	171.04	-	0.08
